# Bridging experiment and theory: a template for unifying NMR data and electronic structure calculations

**DOI:** 10.1186/s13321-016-0120-z

**Published:** 2016-02-09

**Authors:** David M. L. Brown, Herman Cho, Wibe A. de Jong

**Affiliations:** Environmental Molecular Sciences Laboratory, Pacific Northwest National Laboratory, Richland, WA 99352 USA; Physical and Computational Sciences Directorate, Pacific Northwest National Laboratory, Richland, WA 99352 USA; Computational Research Division, Lawrence Berkeley National Laboratory, Berkeley, CA 94720 USA

**Keywords:** Scientific workflow, NMR spectroscopy, Electronic structure theory

## Abstract

**Background:**

The testing of theoretical models with experimental data is an integral part of the scientific method, and a logical place to search for new ways of stimulating scientific productivity. Often experiment/theory comparisons may be viewed as a workflow comprised of well-defined, rote operations distributed over several distinct computers, as exemplified by the way in which predictions from electronic structure theories are evaluated with results from spectroscopic experiments. For workflows such as this, which may be laborious and time consuming to perform manually, software that could orchestrate the operations and transfer results between computers in a seamless and automated fashion would offer major efficiency gains. Such tools also promise to alter how researchers interact with data outside their field of specialization by, e.g., making raw experimental results more accessible to theorists, and the outputs of theoretical calculations more readily comprehended by experimentalists.

**Results:**

An implementation of an automated workflow has been developed for the integrated analysis of data from nuclear magnetic resonance (NMR) experiments and electronic structure calculations. Kepler (Altintas et al. 2004) open source software was used to coordinate the processing and transfer of data at each step of the workflow. This workflow incorporated several open source software components, including electronic structure code to compute NMR parameters, a program to simulate NMR signals, NMR data processing programs, and others. The Kepler software was found to be sufficiently flexible to address several minor implementation challenges without recourse to other software solutions. The automated workflow was demonstrated with data from a $^{17}\hbox {O}$ NMR study of uranyl salts described previously (Cho et al. in J Chem Phys 132:084501, 2010).

**Conclusions:**

The functional implementation of an automated process linking NMR data with electronic structure predictions demonstrates that modern software tools such as Kepler can be used to construct programs that comprehensively manage complex, multi-step scientific workflows spanning several different computers. Automation of the workflow can greatly accelerate the pace of discovery, and allows researchers to focus on the fundamental scientific questions rather than mastery of specialized software and data processing techniques. Future developments that would expand the scope and power of this approach include tools to standardize data and associated metadata formats, and the creation of interactive user interfaces to allow real-time exploration of the effects of program inputs on calculated outputs.

## Background

In the physical sciences, a complex series of steps is often required to relate a theoretical hypothesis to an experimental observable, and *vice versa*. The study of electronic structure by nuclear magnetic resonance (NMR) spectroscopy illustrates the difficulties that can arise with this process. The object of this particular workflow is to transform results from electronic structure simulations into predicted NMR spectra or, in reverse, to extract electronic structure parameters from observed energies and line shapes. This practice can be found in some of the earliest accounts of NMR spectroscopy [1, 2], and it continues to be a valuable and popular approach for elucidating the electronic structure of molecules and crystals.

**Fig. 1:**
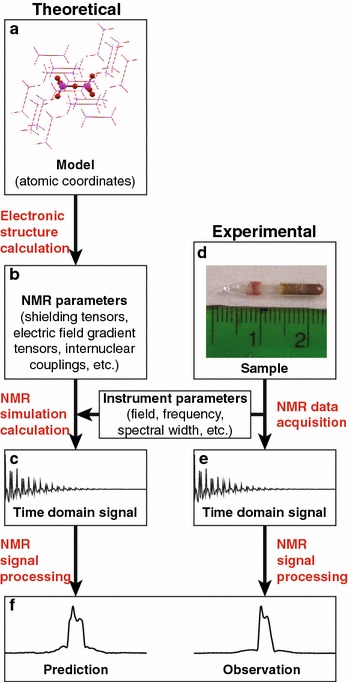
Schematic of NMR data workflow illustrating parallel paths of experimental and theoretical data

A schematic of the forward transformation is shown in Fig. 1. As portrayed in this figure, the workflow encompasses an array of independent computer programs and data inputs, each requiring specialized knowledge for their use. Manual step-wise execution of this workflow is a cumbersome process ill-suited for efficient, interactive fitting of theoretical models and experimental data. Automation of the intermediate steps would greatly expedite the workflow, but in practice requires the merging of software and data from a multitude of instrument makers and electronic structure codes. A further complication is the large variety of NMR experiments and observables an automated workflow might need to accommodate, which necessitates the compilation of a library of experiment-specific simulation programs.

In this paper, we demonstrate how the NMR workflow can be consolidated and simplified with the use of software tools that execute intermediate operations automatically and invisibly. This implementation is part of an effort to enhance the interactivity of experimentalists and theorists that we refer to as the EMSL Experiment/Theory Unification Project (EETUP) [3]. A flexible and modular software architecture has been developed that can accommodate a diverse set of open source software packages, and allows the range of functionality to be expanded with additional modules as new requirements arise. Initial development efforts have been been focused on the analysis of NMR spectra of quadrupolar nuclides, which provide measurements of both chemical shifts and electric field gradients, but more universal applications are possible through the addition of other spectral simulation modules.

## Methods

### Workflow control

**Fig. 2:**
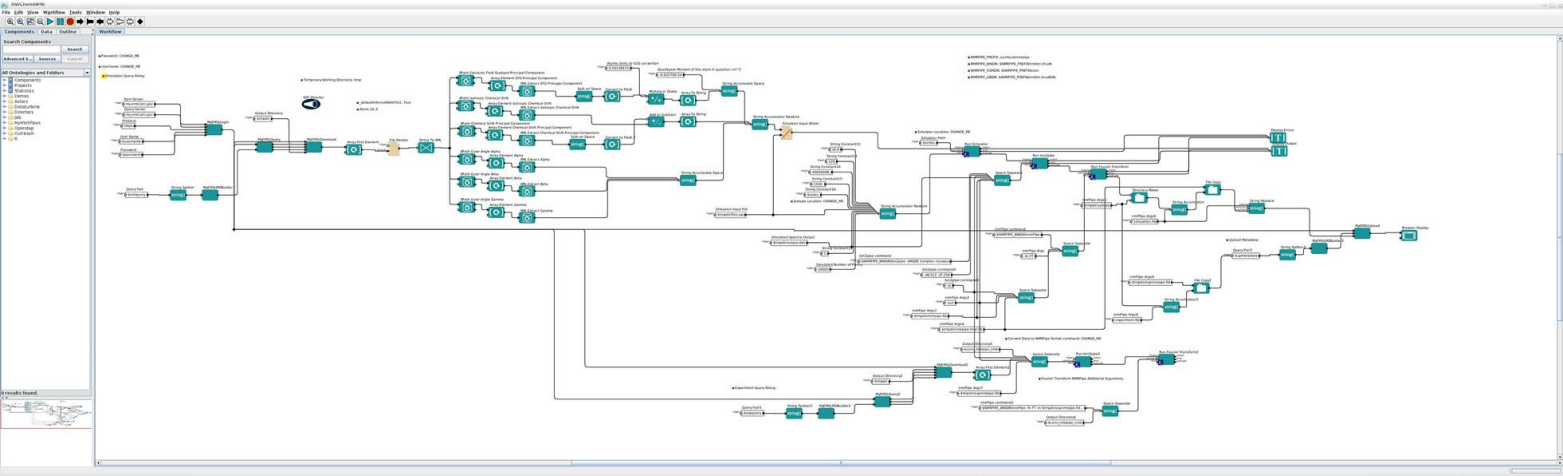
Implementation of workflow as a Kepler process with parallel paths of simulation (*top*) and experiment (*bottom*)

The Kepler Project [4] offers software tools designed to orchestrate complex scientific workflows [5]. In particular, the Kepler interface allows users to create workflow control programs without explicitly writing source code. In addition, Kepler enforces good coding practices in workflow design, including modularity and extensibility of software. The master process we have constructed to manage the workflow in Fig. 1 has been assembled out of tools created by Kepler Project developers. A step-by-step representation of the master Kepler process appears in Fig. 2

The workflow typically spans several different platforms, each dedicated to a specific task: experimental data are acquired with one computer, electronic structure parameters are calculated on another computer, NMR spectra are simulated on a third computer, and so forth. With no single computer controlling the overall workflow, it proves critical to store data in a centralized location. The Active Data Library at our site, MyEMSL, serves as the central access point for EETUP processes. In addition, MyEMSL provides application programming interfaces (APIs) for authentication, querying, and transfers of data. The APIs were abstracted to Kepler Actors through the system SWADL [6–8].

The master Kepler process was programmed in accordance with standard practices to ensure portability to other platforms and adaptability to future needs. Maximal use of actors native to Kepler was made to support essential functions, and a gap analysis was performed to ensure proper exception handling, either with a native actor [9] or an *ExternalExecutionEnvironmentActor* (triple-E) actor that executes custom binary code.

The entry point for the theoretical side of the workflow in Fig. 1a is the calculation of NMR parameters. Electronic structure simulation software relies on sophisticated decisions about basis sets, functionals, molecular structures, atom selections, etc., to be utilized effectively. Due to the specialized and fluid nature of electronic structure codes, the outputs of these programs frequently require checking both for physical reasonableness and for compatibility with subsequent programs in the workflow chain. At present, the complexity of these operations requires direct human interaction with the software and precludes automation within Kepler.

Subsequent steps of the workflow are more readily compatible with automation. The automated part of the workflow in the current implementation begins with the NMR spectral simulation. The simulation code exists as a distinct standalone executable program. Kepler file writer actors prepare the inputs for the code, the triple-E actor executes the simulations, and file reader actors direct the simulation output to the next step of the workflow. The experimental (Fig. 1e) and simulated (Fig. 1c) data are passed through the same NMR signal processing application, and processed in an equivalent manner.

Inputs for the spectral simulation step are entered into the Kepler process as strings, since they are ultimately passed as command line or text input files to the programs performing the calculations. The Kepler process can verify input by converting the values to their appropriate types then back to strings.

### Workflow software and data formats

An array of software choices is available for each step of the workflow. Table 1 lists the applications that were chosen for our current implementation. The applications in this table are open source, in widespread use, and readily extensible. Alternative choices at each step of the workflow can readily be incorporated as user-selectable options within the modular Kepler framework. The software at each step and their input and output data formats are described below.

**Table 1:** Software used in example NMR workflow (refer to Fig. 1)

Task	Program name	Source
Electronic structure calculation	NWChem	Valiev et al. [10]
NMR spectral simulation	Gamma	Smith et al. [11]
Simulated NMR signal processing	NMRPipe	Delaglio et al. [12]
NMR experimental data acquisition	NTNMR	Tecmag, Inc.
	VnmrJ	Agilent
	TopSpin	Bruker
Experimental NMR signal processing	NMRPipe	Delaglio et al. [12]

#### Electronic structure calculation

The electronic structure code used in this implementation, NWChem [10], accepts text inputs and generates Chemical Markup Language (CML) output [13, 14], which is stored on MyEMSL (Fig. 1b). Since CML is a subset of XML, standard Kepler actors for parsing XML are able to extract the data required by the simulation. The use of text files for input and output facilitates the human interaction needed to execute electronic structure codes and interpret results.

Analyses of electronic structure in general require expert decisions by the user on the portion of a molecule or lattice that is to be included in the computation of electronic structure and NMR coupling parameters. This task is performed by manual entry of the coordinates of the selected atoms into the relevant programs, although a graphical user interface could readily be conceived to perform this operation more conveniently and reliably.

#### NMR instrument parameters

NMR instrument data are typically stored in files with proprietary binary formats unique to the instrument’s manufacturer. The $\hbox {C}^{++}$ programs created by us parse current generation data files from NMR instruments manufactured by Agilent, Bruker, and Tecmag. Automated execution of these standalone programs was handled by a triple-E actor in Kepler.

#### Spectral simulation inputs and outputs

The predicted NMR signal is computed from the inputs in Table 2 as a time domain interferogram using the time-dependent density matrix formalism [2]. Simulated spectra in the current implementation are produced by custom $\hbox {C}^{++}$ programs linked against the GAMMA version 4.1.0 NMR simulation environment [11]. Required input data for the simulation are listed in Table 2. Electronic structure and NMR instrument data are obtained as outlined above. The nuclear parameters such as gyromagnetic ratios and quadrupolar couplings represent a relatively small amount of static data, and may be compiled from reference databases and saved in a text file for ease of reading and updating.

**Table 2:** Data entered into workflow simulation program

Source	Parameter
Electronic structure calculation	Shielding tensor principal values ($\sigma _{jj}$)
	Electric field gradient parameters ($V_{zz}$, $\eta _{\mathrm {Q}}$)
	Euler angles relating principal axis systems of $\mathbf {\sigma }$ and $\mathbf {V}$ ($\alpha$, $\beta$, $\gamma$)
	Isotropic shielding value of chemical shift reference ($\overline{\sigma }_{\mathrm {ref}}$)
NMR instrument data file	Spectrometer carrier frequency ($\nu$)
	Frequency at 0 ppm ($\nu _{0}$)
	Spectral digital resolution
Nuclear parameter database	Gyromagnetic ratio ($\gamma$)
	Nuclear spin quantum number (*I*)
	Quadrupole moment (*Q*)
Molecular structure	Atomic coordinates ($\vec {r}_{j}$)
	Internuclear distances and vectors ($\vec {r}_{jk}$)

In addition to the data displayed in Table 2, geometric parameters specifying the orientation of the tensor principal axes with respect to the applied magnetic field direction must be supplied. These data can be in the form of a longitudinal and azimuthal pair of angles representing a single configuration of the tensors, or more commonly, an array of angle pairs representing multiple orientations of the tensors to model a disordered ensemble of nuclear spin systems. To accommodate different models, from a single orientation to an ensemble, the simulation program reads the geometric data from a file and computes and adds spectra for all of the orientations contained in the file.

For correct alignment, the experimental and simulated spectra must be centered at the same chemical shift value, and have equal digital resolutions. The automated process we use to perform the alignment of spectra is explained in Appendix 1.

At present, the outputs of the simulation calculation are stored in a binary packed format directly readable by the processing software selected for our implementation, viz., NMRPipe.

#### Signal processing and visualization

Both the experimental (Fig. 1e) and simulated (Fig. 1c) results are in the form of time domain data, and require processing to obtain frequency domain spectra (Fig. 1f). We have chosen the NMRPipe [12] software package for our first effort to integrate automated data processing in the workflow. NMRPipe is an attractive choice for several reasons: it is open source software in wide use in the NMR community, and provides a comprehensive set of NMR data processing tools. Data analysis in NMRPipe is separated from data display, which greatly simplifies the integration with other processes unconnected with data analysis, such as data uploading and orchestration.

**Fig. 3:**
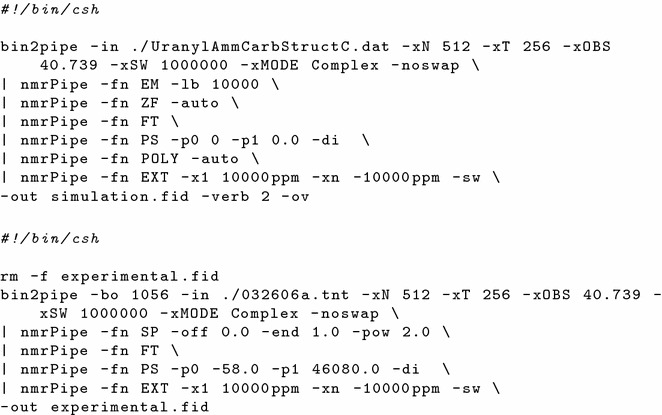
NMRPipe shell scripts used to process simulated (*top*) and experimental (*bottom*) $^{17}\hbox {O}$ NMR data of $(\hbox {NH}_{4})_{4}\hbox {UO}_{2}(\hbox {CO}_{3})_{3}$, with outputs as shown in Fig. 5. These scripts were integrated into the Kepler workflow

NMRPipe recognizes the data formats of all of the major NMR instrument manufacturers, eliminating the need to create programs to translate data to a readable form. Data are passed between individual NMRPipe processes via pipes (see Fig. 3), reading and writing from standard input (*stdin*) and standard output (*stdout*) streams, respectively. Input analysis parameters are entered from command line arguments. To automate this process the *stdout* output stream from one triple-E actor was passed to the *stdin* input stream of the subsequent triple-E actor. Triple-E actors are unable to directly pass data in the preferred format of NMRPipe processes (compressed binary) necessitating the creation of temporary files as the intermediary of data transfers between processes.

The NMRPipe tool, NMRView, is used for final data visualization.

### Case study

A recently published solid-state $^{17}\hbox {O}$ NMR study of $^{17}\hbox {O}$-enriched uranyl salts serves to illustrate the performance of the automated workflow [15]. In this case, experimental results were acquired on a Tecmag, Inc., NMR spectrometer controlled by a computer running a Windows XP operating system. Files stored on this computer were transferred to the centralized data repository, MyEMSL, along with the outputs of the electronic structure calculation performed on the EMSL high performance computer [16].

**Fig. 4:**
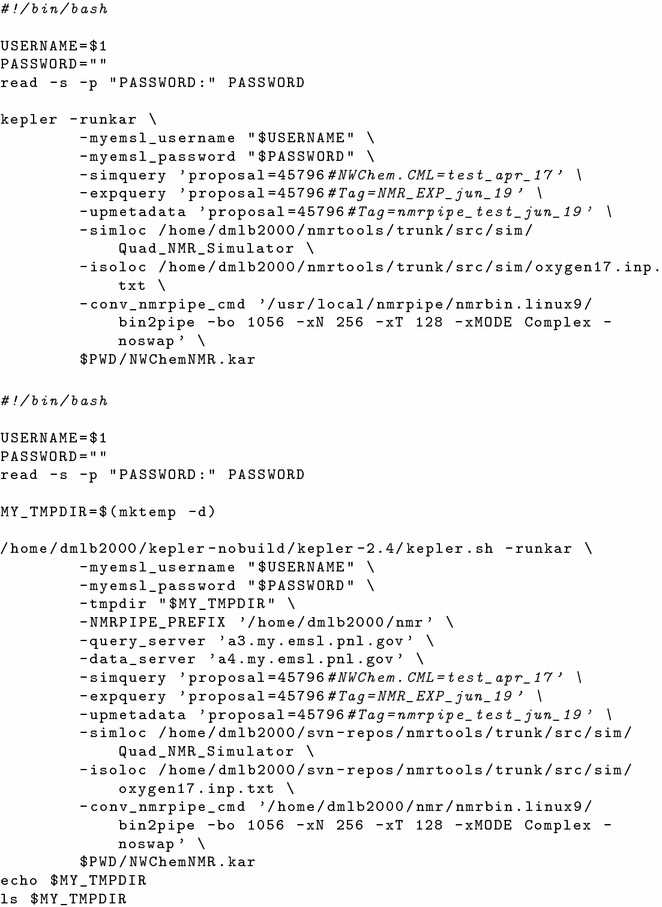
Two scripts for launching Kepler from the operating system command line for convenient analysis when new simulation or experimental results became available

**Fig. 5:**
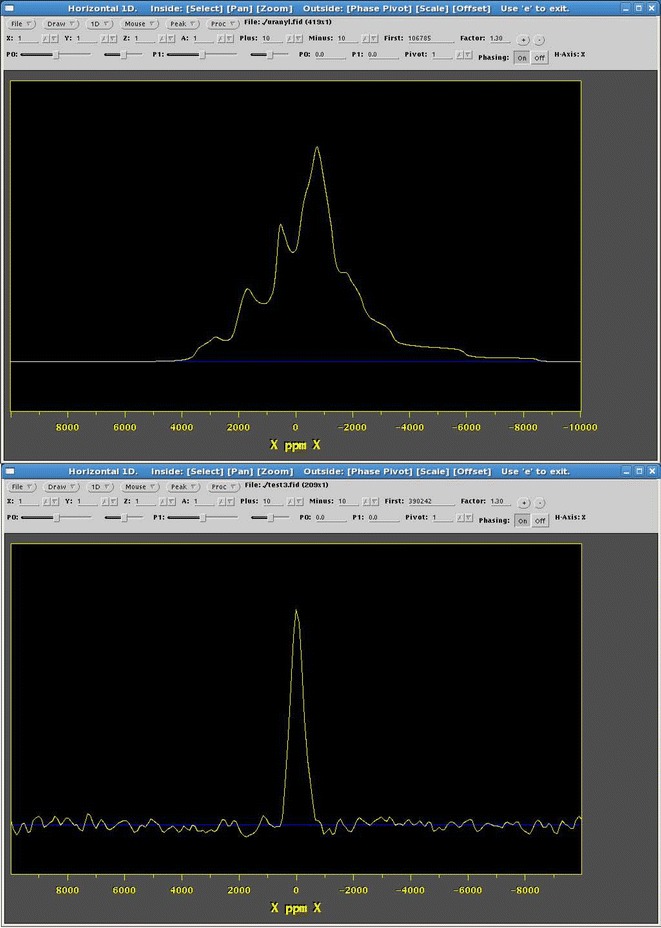
NMRDraw output of simulated (*top*) and experimental (*bottom*) $^{17}\hbox {O}$ NMR spectra of $(\hbox {NH}_{4})_{4}\hbox {UO}_{2}(\hbox {CO}_{3})_{3}$

Upon completion of the data uploads to MyEMSL the automated part of the workflow was initiated on a desktop computer executing the master Kepler process via the scripts shown in Fig. 4. The spectral simulation and processing of the experimental and simulated time domain data were performed on this computer with no further human intervention, and the results directed to MyEMSL (steps $\hbox {B}\rightarrow \hbox {C}\rightarrow \hbox {F}$ and $\hbox {E}\rightarrow \hbox {F}$ in Fig. 1). The final result displayed by the desktop machine appears in Fig. 5, which shows a screen capture of the NMRView window with the predicted (top) and actual (bottom) $^{17}\hbox {O}$ NMR spectra. These spectra may be compared to Figure 5 of reference [15].

The pace of this workflow is slowed by user intervention; the task for the computer at each step may be completed within seconds, but manual data entry and program execution might require several hours of concentrated human effort. By automatically streaming data from computer to computer the Kepler process eliminates the tedious manual steps and can transform the workflow into an instantly interactive operation.

## Conclusion

The implementation described here can serve as a template for the automation of other workflows that blend experimental observables and computational theory. Tools from the Kepler Project provide the capability that allows multiple platforms running sophisticated standalone software to be merged and executed with minimal intervention or expert knowledge on the part of the user. Theory results are made more accessible to experimentalists, and experimental data are more readily interpreted by theorists. All software and documentation developed to date are publicly accessible [3]. Future releases and updates will be made available at this same site. The custom Kepler actors created for this project are also provided at these sites [7], but have not yet been accepted as part of the official Kepler release.

The value of workflow tools will depend to a large extent on their scope and versatility, and in particular their ability to assimilate and process inputs from a wide range of different sources at each step of the workflow. In our current implementation, we have created specialized software tools to read the data formats of the programs in Table 1, but the programming effort and complexity would rapidly increase as more choices were added to the selection in this table. It is clear that expandability of the workflow would be greatly facilitated if data files were standardized to make them universally readable. Standardization of data formats has not been widely implemented [17, 18], but even if adopted at a limited, local level a single unified data format can significantly simplify workflow development.

This implementation would be further improved by automating the creation of NWChem inputs from, e.g., molecular structure data, and starting the NWChem process. Software offered by Avogadro [19] may be superior to Kepler products in this regard and is under consideration as the path for future enhancement. While we foresee no fundamental obstacle to adding this functionality, the specialized knowledge required to select reasonable parameters and estimate computer resources make this a more difficult programming challenge than the ones considered thus far.

Although a central goal of EETUP is the seamless, automatic bridging of theoretical and experimental data, the ability to interrupt and manipulate inputs to the workflow at intermediate steps would add valuable new functionality. Real-time updating of a spectrum as a bond distance or shielding tensor is varied is one conceivable way where such a capability might enhance experiment/theory interactivity.

## Appendix 1: Alignment of experimental and simulated NMR spectra

The automated alignment of experimental and simulated NMR spectra requires a well-defined procedure for relating the field dependent frequency shift computed by electronic structure codes, viz., the nuclear shielding, with the chemical shift measured in NMR experiments. The chemical shift is defined as the scaled difference from the position of a reference signal [20]

1 $$\begin{aligned} \delta _{\mathrm {sample}} = \left( \nu _{\mathrm {sample}} - \nu _{\mathrm {ref}} \right) / \nu _{\mathrm {ref}}, \end{aligned}$$

thus to obtain a chemical shift $\delta _{\mathrm {sample}}$ from a computed shielding result, shielding tensors for the reference compound *and* the compound of interest must both be calculated. Reference compounds for stable NMR-active nuclides have been recommended by the IUPAC [20]. Outputs from two electronic structure calculations are therefore needed by the simulation code, although only the mean of the shielding tensor principal values $\overline{\sigma }_{\mathrm {ref}}$ is utilized from the calculation for the reference compound. These translations were implemented using standard Kepler math actors.

Correct alignment of the simulated spectrum with the experimental result is achieved by specifying simulation parameters that ensure the two spectra have equal digital resolution and the same chemical shift values at the spectrum center. The spectral width need not match, and indeed it can be desirable to have the simulation with a wider frequency range to display features that are not seen in experimental spectrum due to instrument limitations. Spectral alignment exemplifies an operation that involves the interplay of multiple different processes and computers, in this case the instrument computer, the simulation computer, and the machine that performs the electronic structure calculation. Kepler tools were used here for their ability to automate formulaic tasks across platforms, allowing global workflow parameters to be created, manipulated with mathematical actors, and made accessible throughout the workflow.
